# [Retracted] Quercetin promotes the apoptosis of fibroblast-like synoviocytes in rheumatoid arthritis by upregulating lncRNA MALAT1

**DOI:** 10.3892/ijmm.2026.5830

**Published:** 2026-04-15

**Authors:** Fang Pan, Lihua Zhu, Haozhe Lv, Chunpeng Pei

Int J Mol Med 38: 1507-1514, 2016; DOI: 10.3892/ijmm.2016.2755

Following the publication of this paper, it was drawn to the Editor's attention by a concerned reader that certain of the flow cytometric data featured in Figs. 2 and 5, and the control western blot data in Fig. 6A were strikingly similar to data which had been already been submitted for publication in articles that were written by different authors at different research institutes.

Owing to the fact that the contentious flow cytometric and western blot data in the above article were found to be strikingly similar to data that had already been submitted for publication elsewhere, the Editor of *International Journal of Molecular Medicine* has decided that this paper should be retracted from the Journal. The authors were asked for an explanation to account for these concerns, but the Editorial Office did not receive a reply. The Editor apologizes to the readership for any inconvenience caused.

